# A framework for ex-ante evaluation of the potential effects of risk equalization and risk sharing in health insurance markets with regulated competition

**DOI:** 10.1186/s13561-024-00540-4

**Published:** 2024-07-24

**Authors:** Richard C. van Kleef, Mieke Reuser, Pieter J.A. Stam, Wynand P.M.M. van de Ven

**Affiliations:** 1 Erasmus School of Health Policy & Management, Erasmus University Rotterdam, Erasmus Centre for Health Economics Rotterdam (EsCHER), Burgemeester Oudlaan 50, Rotterdam, 3062 PA The Netherlands; 2 National Institute for Public Health and the Environment (RIVM), Antonie van Leeuwenhoeklaan 9, Bilthoven, 3721 MA The Netherlands; 3 School of Business and Economics, Ethics, Governance and Society, Vrije Universiteit Amsterdam, De Boelelaan 1085, Amsterdam, 1081 HV The Netherlands

**Keywords:** Health insurance, Regulated competition, Risk equalization, Risk sharing, Risk selection

## Abstract

Many health insurance markets are organized by principles of regulated competition. Regulators of these markets typically apply risk equalization (aka risk adjustment) and risk sharing to mitigate risk selection. Risk equalization and risk sharing can have various positive and negative effects on efficiency and fairness. This paper provides a comprehensive framework for ex-ante evaluation of these effects. In a first step, we distinguish 22 potential effects. In a second step, we summarize and discuss quantitative measures used for evaluating risk equalization and risk sharing schemes in academic research. To underline the relevance of our work, we compare our framework with an existing framework that was previously used in the Dutch regulated health insurance market. We conclude that this framework is incomplete and uses inappropriate measures. To avoid suboptimal policy choices, we recommend policymakers (1) to consider the entire spectrum of potential effects and (2) to select their measures carefully.

## Background

Many individual health insurance markets are organized by principles of ‘regulated competition’. Examples include the mandatory health insurance schemes in Germany, Israel, the Netherlands and Switzerland, voluntary health insurance schemes in Australia and Ireland, and specific sectors in the U.S. such as Medicare Advantage and the state-based Marketplaces that operate under the Affordable Care Act. In these schemes, consumers can periodically switch insurance plan, which generates competition among insurers. Insurers are financially responsible and are typically given tools to manage healthcare spending such as “selective contracting of healthcare providers” and “choice of provider payment method”. The regulator determines the rules of the game and manages competition. Economic theory suggests that a well-designed combination of ‘competition’ and ‘regulation’ can simultaneously achieve various objectives regarding the efficiency and fairness of health insurance markets [1].

In terms of regulation, all schemes mentioned above include premium-rate restrictions. Although these restrictions enhance fairness (e.g., by making coverage affordable for high-risk people that would otherwise be charged with a very high premium), it is well-known that they also exacerbate selection problems [2]. To mitigate risk selection, regulators typically apply risk equalization (aka risk adjustment) and/or risk sharing. By risk equalization we mean a payment system that (re)distributes funds among insurers using indicators of *expected* cost such as age and health. By risk sharing we mean a payment system that (re)distributes funds among insurers based on *actual* cost. Since the 1980’s, research has led to major improvements of risk equalization and risk sharing schemes (e.g., [3, 4]). Despite these improvements, selection problems remain (e.g., [5–7]), calling for further advances.

(Re)design of risk equalization and risk sharing comes with complex tradeoffs between positive and negative effects on efficiency and fairness. For example, potential negative effects include a reduction of cost control (e.g., when risk equalization and/or risk sharing payments are linked to costs) and a waste of resources due to gaming (e.g., when payments are linked to diagnoses). Potential positive effects include better alignment of insurance products with consumer preferences, improvements of the quality of care, levelling of the playing field, and fewer resources wasted on selection-driven marketing, among others. Studies on the design of risk equalization and risk sharing schemes often focus on a subset of potential effects. For example, most, if not all, researchers and policymakers focus only on the negative effects of risk equalization and risk sharing on efficiency, and not on the positive effects of these instruments on efficiency [8]. The goal of this paper is to provide a framework for ex-ante evaluation of the entire spectrum of potential effects of risk equalization and risk sharing. By considering the full spectrum of potential effects, policymakers will be better informed when making choices regarding the design of risk equalization and risk sharing schemes.

This paper proceeds as follows. Section 2 provides a comprehensive overview of all potential effects of risk equalization and risk sharing on efficiency and fairness. In total, we distinguish 22 potential effects, most of which are positive, and some are negative. This overview provides researchers and policymakers with a *qualitative* framework for ex-ante evaluation of risk equalization and risk sharing schemes. Section 3 summarizes common *quantitative* measures used for ex-ante evaluation of risk equalization and risk sharing schemes. Section 4 demonstrates the relevance of our work by reviewing the evaluation framework that was used in the Netherlands from 2017 to 2022. We find that this framework does not consider all potential effects of risk equalization and risk sharing, and that some of the measures used in this framework are inappropriate. In Sect. 5 we discuss the implications of our work for policy and research.

## Potential effects of risk equalization and risk sharing

To make individual health insurance on a competitive social health insurance market accessible and affordable for high-risk consumers, regulators often implement regulations such as an open-enrollment requirement and premium-rate restrictions for specified basic health insurance products (e.g., [2]). By doing so, the regulator intends to create pooling arrangements with implicit cross-subsidies among heterogeneous risks. For simplicity and clarity of our arguments we assume that the regulator requires community rating per product, which means that an insurer must charge the same premium to all insured who enroll in the same health insurance product. This type of premium regulation is applied in many systems, e.g., the basic health insurance schemes in Germany, the Netherlands, as well as Medicare Advantage in the United States. Other systems include a slightly weaker form of community rating by allowing some limited risk rating according to age and geography, e.g., the basic health insurance in Switzerland and the state-based Marketplaces in the United States. Although premium-rate restrictions help achieve fairness objectives, they also induce a problem: for many consumers the premium strongly deviates from the expected costs of their insurance contract, which is referred to as ‘unpriced risk heterogeneity’. As well-documented in the literature, unpriced risk heterogeneity can lead to risk selection. Inspired by Newhouse [9], we define risk selection as “actions by consumers and insurers that break or intend to break the pooling arrangements”. In Sect. 2.1, we discuss different types of selection actions and their potential effects. Since risk equalization and risk sharing are meant to eliminate selection incentives, reductions of the ‘negative effects of risk selection’ can be seen as ‘positive effects of risk equalization and risk sharing’. These and other effects are summarized in Sect. 2.2.

### Potential negative effects of risk selection

Our definition of risk selection points at two types of selection actions: (1) actions that *intend to break* the pooling arrangements (irrespective of whether these actions indeed break the pooling arrangements) and (2) actions that *break* the pooling arrangements (irrespective of the underlying intention of these actions). Below we discuss both types and their potential negative effects.

#### Actions (by insurers) that intend to break the pooling arrangements

Without further policy measures (such as risk equalization), community-rated premiums would confront insurers with unpriced risk heterogeneity. On average, young and healthy people would be predictably profitable to insurers while the elderly and chronically ill would be predictably unprofitable. These predictable profits and losses confront insurers with ‘incentives to target the young and healthy and deter the elderly and chronically ill’ (or, framed differently: ‘incentives to break pooling arrangements’). In the literature, this form of selection is also referred to as ‘preferred-risk selection’ (e.g., [10]). In most health insurance schemes, risk selection by insurers can take place via the design and marketing of insurance products and/or via other channels such as customer service and supplementary insurance. Examples of selection via product design include structuring coverage in a way that it is relatively unattractive to the chronically ill, e.g., by not contracting providers who have the best reputation in treating or managing specific diseases. Examples of selection via marketing include selective advertising towards healthy people. Selection via customer service can be done by not responding (adequately) to queries from people with specific diseases. And selection via supplementary insurance could mean that insurers charge excessive premiums for supplementary insurance products to groups that are predictably unprofitable for the basic insurance, or do not accept them for supplementary products.^1^ Below, we describe the potential effects of such actions. It is important to emphasize that these effects are independent of whether the actions are successful or not (i.e., whether they eventually break the pooling arrangement). If these actions indeed break the pooling arrangement, additional potential effects enter the stage (see Sect. 2.1.2).

A particularly harmful selection action is when insurers structure their coverage (to the extent they are allowed to do so) in a way that their health insurance products are relatively unattractive to high-risk consumers (e.g., [11–13]). For example, when insurers choose not to contract with providers who have the best reputation for treating specific diseases, patients might not have access to these providers. Moreover, such actions would threaten the level playing field for providers (in terms of getting contracted by insurers) and could even discourage physicians and hospitals from acquiring the best reputation in treating or managing specific diseases. That would be an undesirable, inefficient outcome of a competitive market. In general, a potential effect of selection by distorting coverage is that insurance products are not in line with consumer preferences. Negative effects for high-risk people can also result from distortions of customer service. For example, when insurers aim at deterring high-risk enrollees by delaying answers to their letters and emails, letting them wait during phone calls, and otherwise being impolite to them, the quality of customer service will be suboptimal [14].

Another potential effect of risk selection by insurers is a reduction of cost control. When insurers are confronted with large predictable profits, selection might (be perceived as) a more profitable strategy than improving efficiency in healthcare production. At least in the short run, when an insurer has limited resources available to invest in cost-reducing activities, it may choose to invest in risk selection rather than cost control.

In the presence of unpriced risk heterogeneity, insurers might anticipate adverse selection by consumers and offer a variety of insurance products. For example, these products can differ in terms of coverage, cost sharing and provider network. When one insurer starts offering different insurance products, other insurers must follow if they want to keep attracting predictably profitable people. Such selection-driven product differentiation can seriously reduce the transparency of the insurance market with negative effects on consumer choice and competition.

Finally, investments in risk selection by insurers can be considered a waste of resources because investments exclusively aimed at attracting low risks through risk selection produce no net benefits to society (risk selection is a zero-sum game among health insurers).

#### Actions (by consumers) that break the pooling arrangements

All potential effects listed in Sect. 2.1.1 are independent of whether the underlying selection actions by insurers are successful or not. The extent to which these actions by insurers indeed break the pooling arrangements, depends on the consumers’ reactions. The reason is simple: pooling arrangements will only break when consumers of different risk types sort into different products. If that is the case, additional negative effects will occur. Before we start describing these effects, it is important to emphasize that – in addition to ‘actions by insurers that intend to break the pooling arrangements’ – there exists a whole range of ‘unintended’ actions by insurers that can lead different risk types to sort into different products. For example, it has been shown how a Health Maintenance Organization (HMO) that developed a good reputation for treating chronically ill patients attracted disproportionate shares of predictably unprofitable consumers [15]. In general, any correlation between unpriced risk heterogeneity and consumer preferences regarding dimensions in which insurance products are allowed to vary (e.g., benefits covered, the level of cost sharing, and the quality of provider networks) can lead to the breaking of pooling arrangements. In other words, the breaking of pooling arrangements can result from many combinations of actions by insurers and consumers, irrespective of the underlying intentions. In the literature, sorting of low- and high-risk consumers into different plan types (e.g., low versus high deductible, broad versus narrow provider network) is also referred to as ‘adverse selection’ (e.g., [10, 16]).

Actions by consumers that break the pooling arrangements may cause instability in the insurance market, e.g., when low-risk consumers permanently switch to lower-priced insurance products that are relatively attractive to them [17, 18]. The premiums for the old products will have to rise as they come to be predominantly bought by high-risk consumers. This may then stimulate some high-risk consumers to switch to these new products too, even if the coverage of these products is suboptimal for them. Consequently, premiums for the new products will increase, which may stimulate the low-risk consumers again to switch to new lower-priced products, even if the restricted coverage of these products is suboptimal for them given their risk aversion.^2^ Moreover, since premiums will not only reflect variation in value, but also differences in the insurer’s risk composition, these selection-driven premiums distort the consumers’ price/quality tradeoff and alter competition on efficiency.

Another inefficiency arising from actions by consumers that break the pooling arrangements is the welfare loss due to the potential non-existence of a competitive equilibrium. The continuous exit (bankruptcy) and re-entry of insurers and insurance products come with social costs. Another consequence of actions that break the pooling arrangements is that the cross-subsidies as intended by the regulator are not fully achieved. This may result in unaffordability of health insurance for high-risk consumers. Insurers that specialize in care for undercompensated high-risk patients will have to charge a relatively high premium. In that case high-risk patients can receive good care and good services only if they are able to pay the high premium.

Actions that break the pooling arrangements also distort the level playing field for the insurers. In this context, a level playing field can be defined as a situation in which two insurers who in year *t* have enrolled a different mix of low- and high-risk consumers, but are identical in all other aspects (including, for example, their insurance conditions, their provider network, their coverage of out-of-network spending, their cost efficiency, their premium and their financial reserves), have an identical expected financial result in year *t*. A distortion of the level playing field can be considered unfair to the adversely selected insurers. These insurers must charge a higher premium than their competitors, lose market share and may ultimately go bankrupt, even if they are efficient. In addition, it is hard for insurers to set the premium for the next contract period because they do not know the risk profile of consumers who will sort into their insurance products and how many unprofitable high-risk consumers they must accept during the open enrolment period. This may result in high loading fees to compensate for the risk of being adversely selected, and/or it may result in the bankruptcy of adversely selected insurers.

Finally, in the absence of an effective insurance mandate, low-risk consumers may not buy health insurance because the community-rated premium far exceeds their expected insurance claims. This will increase the premium for those who do buy health insurance. This may further increase adverse selection, resulting in an upward premium spiral. Consequently, the cross-subsidies as intended by the regulator may not be fully achieved, which may reduce the affordability of health insurance coverage. To the extent that risk averse consumers remain uninsured, there will be a forgone welfare gain due to suboptimal risk protection. Moreover, uninsured consumers might not be able to afford expensive treatments in case of serious health problems, especially those with low income [19].^3^

#### Potential negative effects of risk selection

In sum, risk selection may lead to negative effects as summarized in Table 1, some of which relate to technical and allocative efficiency, while others (also) relate to fairness, which is a broad concept that covers normative ideas about solidarity, affordability, cross subsidies, equity, and justice. Fairness may reflect value judgments that differ among individuals and across countries. We consider fairness towards the consumers, the insurers, and the providers of care.

**Table 1:** Potential negative effects of risk selection

Potential effects		Efficiency	Fairness
***Potential effects of actions that intend to break the pooling arrangements:***			
1	A reduction of (the investments in) the quality of care because insurers and providers of care do not strive for obtaining the best reputation for treating people with specific diseases who are unprofitable	x	
2	Absence of a level playing field for providers (since providers with the best reputation in treating specific diseases might not be contracted by insurers)		x
3	Insurance products are not in line with consumer preferences	x	
4	Suboptimal customer service for high-risk consumers	x	
5	Insurers underinvest in cost control when risk selection is (perceived as) a more effective strategy for making profits	x	
6	Distortion of consumer choice and competition due to reduced transparency of the health insurance market because of product proliferation	x	
7	Waste of resources (i.e., resources used for risk selection do not add any social value since risk selection is a zero-sum game)	x	
***Potential effects of actions that break the pooling arrangements***:			
8	Consumers choose the ‘wrong’ insurance product (underinsurance) since premiums of individual insurance contracts are not in line with expected costs	x	
9	Social costs of the absence of a market equilibrium [i.e., excessive exit (bankruptcy) and re-entry of health insurers and health insurance products]	x	
10	Intended income redistribution from low-risk to high-risk consumers is not fully achieved		x
11	Unaffordability of high-value insurance products (which can threaten access to high-quality care and good services, particularly for low-income consumers)		x
12	Absence of a level playing field for insurers		x
13	Bankruptcy of adversely selected – though potentially efficient – insurers	x	
14	Higher loading fees due to the insurers’ risk of being adversely selected	x	
15	Suboptimal risk protection of risk averse (low-risk) consumers who are not *willing* to buy health insurance in the absence of an effective insurance mandate	x	
16	Suboptimal risk protection of risk averse (high-risk) consumers who are willing but not *able* to buy health insurance in the absence of an effective insurance mandate (due to a lack of cross subsidies from low-risk consumers)	x	x
17	Consumers who do not buy insurance (see the above-mentioned potential effects 15 and 16) might not be able to afford expensive treatments in case of serious health problems, especially those with low income		x

### Potential effects of risk equalization and risk sharing

In the presence of open enrollment and premium-rate restrictions for specified basic health insurance products, risk equalization and risk sharing are meant to reduce (the potential negative effects) of risk selection (Table 1). Therefore, reductions of the ‘negative effects of risk selection’ can be seen as ‘positive effects of risk equalization and risk sharing’ (Table 2).

**Table 2:** Potential positive effects of risk equalization and risk sharing (via the reduction of selection)

1	Improvements of the quality of care due to better efforts by insurers and providers to acquire the best reputation for treating patients with specific diseases who previously were unprofitable
2	The playing field for providers is more leveled
3	Insurance products are more in line with consumer preferences
4	Better customer service for high-risk consumers
5	More investments by insurers in cost containment since the potential returns on risk selection decrease
6	A reduction of selection-driven product proliferation which increases transparency on the health insurance market and thereby enhances a value-for-money consumer choice and competition on efficiency
7	Fewer resources spent on selection activities
8	Consumers increasingly choose the ‘right’ insurance product as premium differences between high- and low-value products are less distorted by differences in risk composition across insurance products
9	More stability in the insurance market (resulting in a reduction of the social costs of the absence of a market equilibrium in terms of excessive exit and re-entry of insurers and insurance products)
10	Intended income redistribution from low-risk to high-risk consumers is more fully achieved
11	Increased affordability of high-value insurance products (which improves access to high-quality care)
12	The playing field for insurers is more leveled
13	A lower chance of bankruptcy of (efficient) insurers due to adverse selection
14	Lower loading fees due to a reduction of the risk of adverse selection

If there is no effective mandate to buy health insurance, the impact of risk equalization and risk sharing on the last three negative selection effects in Table 1 (number 15–17) depends on how risk equalization and risk sharing payments are financed [20]. If these payments are financed ‘internally’, i.e., via the premiums (as e.g., in the ACA marketplaces in the United States and in the voluntary health insurance markets in Ireland and Australia), the premium for low-risk people might increase. More specifically, when low-risk consumers are concentrated in specific insurance products (e.g., with a high-deductible), risk equalization and risk sharing are likely to drive up the premiums of these products. This may stimulate a new group of low-risk consumers not to buy health insurance and exacerbates the negative selection effects 15–17 in Table 1. ‘External’ financing of risk equalization and risk sharing payments, e.g., via taxes or mandatory contributions, will reduce the premium, both for high-risk and low-risk consumers, because they function as a subsidy to the market. This may stimulate some uninsured low-risk consumers to buy insurance, which reduces the negative selection effects 15–17 in Table 1. In the case of a mixture of internal and external funding of the risk equalization and risk sharing payments, the effect depends on the weights given to the internal and external funding.

In practice, the positive effects of risk equalization and risk sharing come with a price, not only because of the direct costs of data collection and preparation but also because of the indirect costs of reduced incentives for cost containment. First, state-of-the-art risk equalization schemes have diagnoses/cost-based risk adjusters which can reduce the insurers’ incentives for cost containment, because a reduction of healthcare utilization or healthcare expenses may result in lower future equalization payments. The stronger the endogenous link between utilization/spending and (future) risk equalization payments, the weaker will be the incentives for insurers to control prices and volumes of care. Second, in settings where risk equalization formulas are being regularly updated and modified, insurers might try to influence the regulator with the goal to include or remove specific factors (or make other changes to the formula) that might be beneficial for them. Such efforts might distract insurers from improving efficiency.

Risk equalization schemes may also lead to perverse incentives. For example, insurers and/or the contracted providers may provide unnecessary services to code a diagnosis, upcode diagnoses to more serious conditions, or (fraudulently) distort information reported to the regulator that is used for payment purposes.

Risk equalization payments based on prior diagnoses might also reduce incentives for prevention, both in terms of primary prevention (i.e., efforts to prevent diseases before they occur) and tertiary prevention (i.e., helping people manage long-term, often-complex health problems). The reason is that if an insurer improves the health of its enrollees by effective prevention, this may result in lower future equalization payments. (For counter arguments, see [2]).

Like risk equalization, risk sharing also comes with a price. The ex-post cost-based payments to the insurers *ceteris paribus* reduce the insurers’ incentives for cost containment and prevention. The size of this effect depends on the amount and the funding of the ex-post compensations and on other design aspects of a risk sharing scheme (e.g., [21, 22]). The *net* effect of risk sharing on efficiency is the sum of this negative effect and the *positive* effects mentioned in Table 2, and can be either positive or negative.

The potential negative effects of risk equalization and risk sharing are summarized in Table 3.

**Table 3:** Potential negative effects of risk equalization and/or risk sharing

18	Cost of data collection and preparation
19	Reduction of cost control
20	Unnecessary services to code a diagnosis
21	(Fraudulent) change of the diagnostic code (e.g., upcoding of diagnoses to more serious conditions) and other distortion of the information reported to the regulator
22	Reduction of prevention

Although the magnitude of the effects in Table 3 might vary across settings, these effects are largely inherent to risk equalization models with endogenous risk adjusters (i.e., diagnoses- or cost-based risk adjusters). In addition to these inherent effects, there can also be negative effects due to shortcomings of risk equalization methodology. For example, estimation of risk equalization models is often based on observed spending, either from the current year or a prior year. Variation in observed spending, however, does not only result from differences in “true health risk” but also from differences in “efficiency” across insurers and providers as well as “under/overutilization” by consumers. In general, risk equalization models are supposed to compensate exclusively for variation in health risk. When risk equalization models are estimated on observed spending, however, payment weights might (partly) reflect other sources of spending variation. For example, to the extent that individuals with a specific risk adjuster flag (e.g., a certain age class or DCG) are concentrated in a specific insurance plan, the payment weight for that risk adjuster might capture the cost (in)efficiency of that plan compared to the other plans in the market. Consequently, this cost (in)efficiency is (partly) compensated for by the risk equalization system, which reduces the plan’s incentives for cost control [23]. Another example is the scenario in which specific disease groups or geographical areas are underserved. When a risk equalization model includes risk adjusters that explicitly flag these disease groups or geographical areas, the payment weights for these risk adjusters will capture the inefficiency (and thus be too low). This way, the risk equalization model might perpetuate existing inefficiencies [2]. For other examples of how estimation of payment weights using observed spending might lead to suboptimal outcomes (and for solutions), we refer to [23].

In sum, risk equalization and risk sharing come with positive and negative potential effects that require a complex trade-off. Tables 2 and 3 can be used by policymakers as a qualitative assessment framework for making decisions on (re)design of their risk equalization and/or risk sharing scheme. It is important to note that the occurrence and size of these effects depend on the institutional context (e.g., the choice options for consumers, and the efficiency and selection instruments for insurers) and design features of risk equalization and risk sharing. For example, the negative effects of risk selection (and thus the positive effects of risk equalization and risk sharing) might be more extensive as the scope for risk selection (in terms of possible selection actions by consumers and/or insurers) is larger [24].

## Quantitative measures for ex-ante evaluation of risk equalization and risk sharing

This section summarizes the quantitative measures that have been developed and used in the literature for ex-ante evaluation of risk equalization and risk sharing schemes. We also discuss how these measures relate to the potential effects discussed in Sect. 2.2. We first summarize measures related to risk selection (Sect. 3.1) and then discuss measures related to gaming and cost control (Sect. 3.2). We focus on so-called ‘ex-ante’ measures, i.e., measures used to *simulate* incentives and to *predict* actions and/or effects. Papers in this stream of literature include studies on the development and evaluation of risk adjuster variables (e.g., [25–27]), studies on the evaluation of ‘payment fit’ under existing payment systems (e.g., [5, 28–30]) and studies on the design and evaluation of risk sharing modalities (e.g., [6, 21, 22, 31, 32]). Given their ex-ante nature, these studies differ from the stream of literature on ex-post measurement of *actual* actions and effects, e.g., in terms of risk selection and gaming (e.g., [14, 33–38]).

### Ex-ante measures related to risk selection

Risk equalization and risk sharing are meant to eliminate selection problems by compensating insurers for variation in *expected* costs of insurance contracts that is *not* allowed to be explicitly reflected in premium variation (‘unpriced risk heterogeneity’). When risk equalization and/or risk sharing perfectly compensate for unpriced risk heterogeneity, risk selection will be absent. Below we discuss some common measures that have been used to quantify the extent to which risk equalization and risk sharing compensate for unpriced risk heterogeneity. In line with Sect. 2, we make a distinction between ex-ante measures that relate to ‘actions by insurers that are intended to break the pooling arrangements’ (Sect. 3.1.1.) and ex-ante measures that relate to ‘the breaking of pooling arrangements’ (Sect. 3.1.2).

#### Ex-ante measures related to actions that intend to break the pooling arrangements

In the academic literature, many ex-ante measures have been developed and applied that relate to selection by insurers. For a comprehensive overview, see [39, 40]. We categorize existing measures into three groups: (1) measures of explanatory power, (2) measures of incentives and (3) measures of expected actions and their effects. Below, we describe these categories and highlight the most common measures in each category.

##### Measures of explanatory power

The most applied measure in ex-ante evaluations of risk equalization models is the R-squared. This measure indicates the proportion of variance in medical spending that can be explained by the independent variables (i.e., risk adjusters) of the risk equalization regression model. Recent studies have applied the R-squared as a measure to quantify the proportion of variance in medical spending explained by the *entire* payment system, which – in addition to risk equalization – can consist of risk sharing and/or premiums (e.g., [22, 40, 41]). Although the R-squared can be informative, the link between this measure and the effects listed in Sect. 2 of this paper is questionable. The reason is three-fold. First, the R-squared includes a quadratic weighting of errors. Although large errors may be more problematic than small errors, it is not obvious that quadratic weighting is better for evaluations of risk equalization than alternative forms of weighting. To overcome this shortcoming, some studies have (also) applied linear measures of explanatory power such as Cumming’s Prediction Measure (CPM) and the Mean Absolute Prediction Error (MAPE). Second, the R-squared (as well as the CPM and MAPE) summarizes gaps (errors) between individual-level predicted costs (or: revenues) and *actual* costs. Selection incentives, however, result from gaps between revenues and (insurer or consumer) *expected* costs. This is an important shortcoming since the lion’s share of individual-level variance in spending is not *predictable* and thus cannot be anticipated on by insurers and consumers. Third, related to the previous points, incentives for risk selection do not monotonously decrease with a higher R-squared (i.e., an increase in R-squared does not necessarily imply a reduction of the incentives for risk selection). This can be illustrated with the following example: a payment system with 50% proportional risk sharing (and without risk equalization) has an R-squared of 0.75 and a CPM of 0.50 while systems with state-of-the-art prospective risk equalization have an R-squared and CPM that are much lower, somewhere in the range of 0.30–0.35. Despite the lower R-squared and CPM, however, these prospective risk equalization models are likely to better mitigate risk selection incentives than 50% proportional risk sharing.^4^

##### Measures of incentives

Many studies have recognized the shortcomings of the R-squared, CPM and MAPE and (also) apply measures that more directly relate to *incentives* for insurers to engage in risk selection. These measures include under/overcompensation and predictive ratios for groups of interest from a prior period (e.g., [5, 29, 30, 42]). If the groups are large enough, the actual average costs per person in such a group can be interpreted as the ‘expected/predicted costs’ for a person in that group.^5^ Whereas under/overcompensations show the monetary value of the difference between predicted costs and actual costs for subgroups, predictive ratios show the ratio of predicted costs and actual costs for subgroups. If these measures are calculated for groups that are (potential) targets of specific selection actions, they meaningfully indicate the incentives for insurers to engage in these actions. For alternative measures to do so, see [28, 43]. Other meaningful measures of incentives are ‘predictiveness and predictability’, which together indicate the insurers’ incentives for service-level distortion, i.e., the incentives to deviate from the socially optimal allocation of resources across medical services [44].

##### Measures of expected actions and their effects

Although group-level under/overcompensation and predictiveness and predictability relate directly to incentives for selection by insurers, they do not capture the ‘expected *effects*.’ To go beyond incentives, one must have a clear idea about how incentives feed into ‘actions’ (whether to engage in a specific selection action or not) and how actions feed into ‘effects’. Only a few studies have explored going beyond incentives. The most prominent work in this direction are the papers on ‘service-level distortion’ ( [45–47]). These papers rely on a model of insurer behavior. By making assumptions on the objective of insurers (profit maximization), the level on which insurers ‘take action’ (decisions on how to allocate premium revenues to healthcare services) and the insurers’ expectation of consumer behavior (how consumers value service-level allocations), the model allows predicting how much insurers will spend on specific services under a given payment system. When the ‘socially optimal’ service-level allocations are known, this model allows for indicating the expected ‘welfare losses’ under alternative payment models.

Other studies follow a more pragmatic approach by applying some form of non-linear and/or asymmetric weighting of group-level under/overcompensations. One example includes ignoring small under/overcompensations based on the assumption that insurers are unlikely to act on small under/overcompensations given the costs of risk selection [48]. Another example includes giving more weight to undercompensation of unhealthy groups than undercompensation of healthy groups based on the assumption that undercompensation of unhealthy groups is more likely to result in quality skimping and therefore comes with worse welfare effects than undercompensation of healthy groups [49]. A meaningful application of non-linear and asymmetric weighting, however, requires consensus about (1) the groups or services of interest and (2) the weighing of under/overcompensations for these groups/services given the likelihood and potential effects of specific selection actions.

#### Ex-ante measures related to the breaking of pooling arrangements

If all insurers are equally successful with their selection actions, the distribution of low- and high-risk individuals might be roughly similar across products. Under such circumstances, the potential effects of risk selection are ‘limited’ to the effects 1–7 in Table 1. However, as soon as low-risk and high-risk people start sorting into different products, the negative effects 8–14 in Table 1 may occur. One measure to evaluate the extent to which a payment system compensates for (potential) risk variation across products has already been discussed in Sect. 3.1.1: group-level under/overcompensation. This measure does not just indicate the incentives for insurers to select in favor or against specific groups (Sect. 3.1.1.2), but also the under/overcompensation of insurance products under *extreme* sorting patterns. For example, the Dutch risk equalization model of 2016 overcompensates people who reported a (very) good health in the prior year by 187 euro per person per year and undercompensates the complementary group by 512 euro per person per year [5]. These figures imply that when the two groups would perfectly sort into different products, the mean costs of these products *net of risk equalization* would differ by 699 euro per person per year (which is about 28% of the average annual health expenses per person). In practice, however, such perfect sorting is unlikely and should be regarded as one end of the spectrum, with the other end being ‘no sorting’.

Measures of group-level under/overcompensation indicate the extent to which pooling arrangements could break, but do not directly predict the expected effects. For a meaningful prediction of effects, a model is needed on insurer and consumer behavior. On the insurer side, such a model needs valid estimations (or at least plausible assumptions) on how risk equalization and/or risk sharing payments translate into premiums. More specifically, researchers must have a clear idea of how under/overcompensation of a population with a certain insurance product will be reflected in a higher/lower premium for that product, ceteris paribus. With imperfect competition the ‘pass through’ of under/overcompensation into premiums may be incomplete. Indeed, a pass-through rate of about 50% has been found in Medicare Advantage [50]. Since the level of competition is likely to differ across countries and markets, estimations of insurer behavior in one setting cannot be extrapolated to other settings. On the consumer side, prediction models of consumer sorting need valid estimations of demand for (specific) insurance (products) and the correlation of demand with expected costs. With such a model, researchers can predict the sorting equilibrium in health insurance markets and the welfare loss resulting from inefficient sorting due to adverse selection (e.g., [51–54]). Moreover, such a model allows predicting and evaluating the sorting patterns under alternative risk equalization and/or risk sharing designs (e.g., [55–57]). In terms of welfare effects, existing studies have focused on the ‘forgone welfare gain’ from underinsurance (in terms of not buying insurance or buying too little insurance). These studies have not explicitly considered the other potential effects of the breaking of pooling arrangements as listed in Table 1 of this paper.

### Incentive measures for cost control and gaming

Risk sharing systems provide insurers with payments based on *actual* cost. Such a link between costs and payments *ceteris paribus* reduces the incentives for insurers to control costs, i.e., it reduces the ‘power’ of the payment system [58]. A pragmatic approach for indicating power under risk sharing systems is to look at the fraction of overall revenues that is allocated via risk sharing [6, 32]. A more sophisticated approach is to simulate the effect of a price increase for an average risk portfolio on the payments from a high-risk pool [22, 59].

Like risk sharing, risk equalization can create a link between costs/utilization and payments too. If risk equalization is solely based on exogenous risk adjusters (i.e., risk adjusters that cannot be influenced by insurers, such as age, gender, and socioeconomic variables) it has maximum power, i.e., 1.0. But if risk equalization is (partly) based on endogenous risk adjusters (i.e., risk adjusters that can be influenced by insurers, such as indicators based on prior spending and diagnoses) power falls below 1.0. It is important, however, that even in the case of a power smaller than 1.0, the *total* effect of risk sharing or endogenous risk adjusters on efficiency may be *positive* due to their 14 potential positive effects on efficiency (Table 2). We are not aware of any study that takes these 14 potential positive effects on efficiency into account explicitly.

When it comes to gaming, incentives for insurers to engage in upcoding or inducing demand for unnecessary care can be indicated by comparing the ‘costs’ of a treatment that leads to a risk adjuster flag and the payment weight associated with that risk adjuster flag. For example, Van Kleef & Van Vliet [60] compare the cost of various medical devices for patients (such as prosthesis and tube feeding equipment) with the insurers’ costs of these devices. For all fourteen devices included in the Dutch risk equalization model of 2008, the payment weight far exceeded the insurers’ costs, implying substantial incentives for gaming.

The aforementioned measures of cost control and gaming are pure incentive measures and do not predict the expected *actions* and/or *effects*. For the prediction of actions and effects, researchers must know how incentives feed into behavior. This requires a clear idea of all potential ‘actions’ that insurers could take. In addition, researchers must have valid estimations or plausible assumptions about the link between incentives and behavior. For example, it is questionable whether the link between power and cost control efforts is linear. It could well be that above a certain level of power insurers might actively invest in cost control, but that below that level they will abruptly stop these investments, implying that cost control efforts are a non-linear function of the power of a payment system. As far as we know, no study has gone beyond indicating incentives for cost control and gaming by predicting actions and effects.

### Conclusion

This section has provided an overview of quantitative measures used in the academic literature for ex-ante evaluation of risk equalization and risk sharing schemes (or ex-ante evaluation of selection incentives more generally). Based on this overview, two important observations can be made. First, in the light of the potential effects of risk equalization and risk sharing, some measures are more informative than others. Therefore, the choice of measures should be made carefully, i.e., should link to the potential effects that are relevant in the context of interest. Measures of explanatory power, such as the R-squared, CPM and MAPE, are hardly informative since the link between these measures and the potential effects of risk equalization and risk sharing is rather weak. Measures of under/overcompensation for groups of interest are much more informative, either for indicating the incentives for insurers ‘to break pooling arrangements’ or for indicating the extent to which ‘the pooling arrangement can break’ under a risk equalization and/or risk sharing scheme. Second, measures of under/overcompensation have their limitations too since they do not directly indicate the expected effects. In the literature only two types of measures have been developed that allow for prediction of effects by using economic models of how incentives (are expected to) feed into behavior and effects: the measures of service-level distortion (e.g., [45]) and the measures of forgone welfare gain due to underinsurance of low-risk consumers (e.g., [52]).

## Which potential effects are considered in policy and how? The case of the Netherlands

Our considerations above imply that – to avoid suboptimal policy choices – policymakers should (1) consider the entire spectrum of potential effects of risk equalization and risk sharing, and (2) select their quantitative measures carefully. To underline the relevance of our work, this section reviews the official evaluation framework used for ex-ante evaluation of risk equalization and risk sharing in a specific policy context: the regulated health insurance market for curative care of the Netherlands. This scheme is based on the model of regulated competition. Important aspects of competition include a free consumer choice of health insurer and a toolkit for insurers to improve efficiency of care such as ‘the possibility to selectively contract with providers of care’ and ‘the option to apply innovative provider payment methods’ such as value-based payments. Important regulatory features include an insurance mandate for consumers, an open enrollment requirement for insurers, community-rating per insurance product and a system of risk equalization and risk sharing. In theory, all potential effects of risk equalization and risk sharing described in Sect. 2 of this paper are relevant in the Dutch scheme, except potential effects 15–17 due to the presence of an effective insurance mandate.

Below, we first describe the risk equalization policy and research cycle in the Netherlands (Sect. 4.1). After that, we discuss the potential effects that are considered quantitatively (Sect. 4.2) and qualitatively (Sect. 4.3). Section 4.4 summarizes our main observations.

### Policy & research cycle in the Netherlands

The annual policy and research cycle for maintaining and improving the risk equalization model starts with a research agenda and ends with a recommendation to the Minister of Health on the preferred modifications. The policy and research cycle in the Netherlands is rather unique, in the sense that there is a strongly committed Expert Committee supervising all stages of the research cycle. This Expert Committee consists of about 70 representatives from research institutes, health insurers as well as governmental bodies like the Ministry of Health, the Ministry of Finance, and the Healthcare Institute (which is responsible for transferring the equalization payments to the insurers). The chairperson and the secretary come from the Ministry of Health.

The annual policy and research cycle for the risk equalization model of year *t* starts in July of year *t*-2 with the formulation of a research agenda. The research projects start in the fall and are carried out in two phases. In the first phase, separate research projects are conducted to explore potential model modifications. These projects typically focus on the refinement and updates of existing risk adjusters but sometimes also involve the development and evaluation of completely new risk adjusters. These separate research projects are supervised by a selection of the Expert Committee. Once these projects are completed, the second phase starts, i.e., a simulation of the effects of all proposed model modifications from the separate projects combined. This second phase takes place in the summer of year *t*-1 and uses the most recent data available. Based on the results of this research, the Expert Committee advises the Minister of Health on the preferred (combination of) modifications of the risk equalization model for year *t*.

To evaluate the effects of (potential) model modifications the Expert Committee has developed an ex-ante Evaluation Framework [61]. This framework functions as a guideline for researchers and the Expert Committee to objectivize policy recommendations. This Evaluation Framework consists of quantitative measures for ex-ante evaluation of alternative risk equalization models as well as more qualitative measures or arguments to consider in decision making, like cost control incentives, complexity, validity, and data-reliability. Below, we focus on the version of this Evaluation Framework that was used from 2017 to 2022.^6^

### Risk equalization and risk sharing effects considered quantitatively

Research on the calibration, evaluation and modification of the Dutch risk equalization model applied in year *t* is typically based on a dataset with spending from year *t*-3 and individual risk characteristics from the period *t*-8 to *t*-3. For details, see [62]. Various measures are used to evaluate the existing model and potential improvements.

#### Quantitative measures to evaluate predictive performance

Since the introduction of risk equalization in the Netherlands in 1993, the R-squared has been one of the most-used measures to evaluate the performance of the risk equalization model. All research projects have reported the R-squared on the level of individuals and the level of insurer portfolios. Since 2015, the Cumming’s Prediction Measure (CPM) has also been reported. Another common measure is the mean absolute prediction error (MAPE), which is reported on the individual level, insurer level and subgroup level (across all combinations of risk adjusters included in the risk equalization model; in 2017: 1.85 million groups in total). For each individual insurer the mean per person prediction error is anonymously reported.

In 2017, a risk equalization symposium led to a revision of the Evaluation Framework. Position papers of the symposium and debates in the parliament expressed concerns about risk selection against people with a chronic disease and in favor of healthy people [63]. To better monitor selection incentives, the following subgroups were added to the Evaluation Framework: (1) consumers in the bottom-15% of the spending distribution three years ago and (2) those in the top-15% of the spending distribution three years ago.

**Table 4:** Quantitative measures included in the Dutch evaluation Framework for evaluating the risk equalization model (version: 2017–2022)

Level of measurement	Quantitative measure
Individuals	R-squared
	Cummings Prediction Measure
	Mean Absolute Prediction Error (MAPE)
Subgroups	Mean weighted absolute prediction error over all combinations of risk adjusters included in the risk equalization model (weighted with the number of insured years in each combination)
	Mean per person prediction error for somatic care for the following groups: • Bottom-15% of spending distribution for somatic care three years ago • Top-15% of spending distribution for somatic care three years ago • Spending for home care in the prior year > 0
	Mean per person prediction error for mental care for the following groups: • Bottom-25% of spending distribution for mental care in the prior year • Top-1% of spending distribution for mental care in the prior year • Top-3% of spending distribution for mental care in the prior year • Top-5% of spending distribution for mental care in the prior year • Spending for mental care in the prior year > 0 • Spending for mental care in the prior year = 0
Insurer portfolios	R-squared
	Mean weighted absolute mean per person prediction error over insurer portfolios (weighted with the number of insured years per portfolio)
	Mean per person prediction error per individual insurer (anonymously)
	Range (difference between min and max) of mean per person prediction error
	Mean weighted absolute change in prediction error when moving from model A to B (weighted with the number of insured years per portfolio)

Source: [61]

Another measure that is often calculated in ex-ante evaluations of the risk equalization model is the sum of funds that the model redistributes across individuals in the total population. This amount (and development in this amount over time), was sometimes used in letters to the Parliament as a measure of performance of the risk equalization model.

It is interesting to note that six measures in the Dutch Evaluation Framework are *not* considered in academic research (see Sect. 3). This holds for the first measure at the group level and all measures at the insurer level in Table 4. All these six measures are hard to interpret and are invalid measures for quantifying selection incentives. A problem with the mean weighted absolute prediction error over all combinations of risk adjusters is that it can substantially underestimate incentives for risk selection. For example, after adding all interaction terms to a poorly performing risk equalization model, the mean weighted absolute prediction error over all combinations of risk adjusters will be zero, while substantial selection incentives can remain.

A problem with the measures at the insurer level is that the outcomes of these measures heavily depend on the distribution of risk types across insurers’ portfolios and on the cost structure in these portfolios. For example, consider the worst risk equalization formula with for each person the predicted expenses equal to the mean per person expenses in the population. Such a risk equalization model has maximum incentives for risk selection. Nevertheless, if the risk composition and cost structure are identical across the insurers’ portfolios, the R-squared is 1.0 and the MAPE is zero. So, the R-squared and the MAPE at the insurer level are no valid indicators of *selection incentives*. Also, the range of the mean per person prediction error across insurer portfolios is not a good indicator of a level playing field, because under the same assumptions this range is zero for any risk equalization formula. In addition, the risk equalization model of year *t* is evaluated by using the dataset with spending from year *t*-3. Given that, in the Netherlands, about 6% of the population switches during the annual open enrollment period, the risk composition of insurers’ portfolios in year *t* (i.e., 3 years later) might have substantially changed. And in the case of ‘perfect’ risk equalization, the (range of the) mean per person prediction error reflects the different cost structures of the insurers and may not be zero.

Although the mean per person prediction error at the insurer level is no valid indicator of selection incentives, it can function as a signal of potential shortcomings of risk equalization and risk sharing. In case of a substantial mean per person prediction error for a specific insurance plan, the regulator could review the design and marketing of that plan as well as the group of people that sort into that plan. Such information might help the regulator identify relevant risk factors that are insufficiently compensated by risk equalization and risk sharing.

#### Quantitative measures for cost control

In addition to the measures mentioned in Table 4 (which evaluate the fit between predicted and actual spending), negative effects of risk equalization and risk sharing on cost control have always played an important role in the evaluation of the risk equalization model and in decisions for model modifications. The Evaluation Framework also pays a lot of attention to incentives for cost containment, both in choosing the risk adjusters as well as in the design of risk adjusters.

The effects of risk equalization and risk sharing on cost control are mainly evaluated by qualitative considerations. The framework states, for instance, that a risk adjuster based on health status information is always preferred over a risk adjuster based on actual (prior) spending. The only quantitative measure included in the Evaluation Framework is the so-called ‘repayment ratio’, which is closely related to the power measure mentioned in Sect. 3. This measure takes the ratio of the ‘payment weight for risk adjuster *k*’ over the ‘mean per person cost of the treatment (or pharmaceutical) that leads to a flag for risk adjuster *k*’.

### Risk equalization and risk sharing effects considered qualitatively

Some of the criteria in the Evaluation Framework are not evaluated via quantitative measures but via qualitative considerations. Apart from the negative effects of risk equalization and risk sharing on cost control incentives, the considerations mainly relate to implementation issues like complexity and transparency of the model and the validity and measurability of the risk adjusters. As mentioned above, the effect of risk equalization and risk sharing on cost control are mainly considered in a qualitative way. These considerations have led policymakers to prefer health-based over cost-based risk adjusters, and prospective over concurrent morbidity indicators. One clear and substantive example of how gaming (or upcoding) incentives play a role in risk equalization design are the thresholds of (e.g., 180) defined daily doses applied to the Pharmaceuticals Cost Groups to prevent ‘gaming’, e.g., by prescribing small doses of drugs.

These considerations of cost control concerns have also been fundamental in the discussion on ex-post risk sharing. The political believe has been that financial responsibility for insurers promotes cost control, which contributes to sustainable health spending. Explicit quantitative measures to assess incentives or effects of risk sharing were never analyzed or used.

### Conclusion

This section has discussed the framework used for ex-ante evaluation of risk equalization and risk sharing in a well-developed policy context: the regulated health insurance market for curative care of the Netherlands. We conclude that the Evaluation Framework used in the annual policy cycle in the period 2017–2022 is incomplete and partly invalid. First, the framework does not look at the entire spectrum of potential effects mentioned in Sect. 2.2. For example, none of the 14 potential positive effects in Table 2 are explicitly considered in the Evaluation Framework, neither qualitatively nor quantitatively. Second, the set of quantitative measures in the Evaluation Framework is very limited. While the ‘mean per person prediction error for subgroups’ is a meaningful indicator of selection incentives for insurers, the set of groups for which this measure is calculated is very limited. Important groups – such as groups with specific diseases that insurers can select against – are missing. Third, the Evaluation Framework heavily relies on measures of statistical fit such as the R-squared and CPM (calculated at the individual level). As discussed in Sect. 3 of this paper, such measures are hardly meaningful for indicating the potential effects of risk equalization and risk sharing. Finally, at the subgroup and insurer-portfolio level, the Evaluation Framework includes six measures that are not considered in academic research (see Sect. 3). All six measures are hard to interpret and are no valid measures to assess the impact of risk equalization on the incentives for risk selection.

## Conclusion and discussion

This paper has developed a framework for evaluating the potential effects of risk equalization and risk sharing in regulated competitive health insurance markets. More specifically, we have provided an overview of the potential effects (Sect. 2) and summarized the ex-ante measures that have been developed and applied in the literature to quantify (some of the) potential effects (Sect. 3). Along the way, we have made two crucial observations. First, the potential effects are numerous and diverse: in total, we distinguished 22 potential effects, most of which relate to efficiency and some (also) to fairness (see Sect. 2.2.). Fourteen of these potential effects work in a positive direction. Five potential effects work in a negative direction and relate to the direct cost of risk equalization and risk sharing or the indirect costs due to perverse incentives. Three potential effects relate to selection by consumers in/out the market (which are not relevant for markets with an effective mandate) and work in a positive or negative direction, dependent on how risk equalization and risk sharing payments are financed. Second, the quantitative measures developed and applied in the academic literature are not perfectly aligned with the set of potential effects. While the literature offers a wide range of quantitative measures, some measures are hardly informative. Therefore, the choice of measures should be made carefully. Moreover, we find that most of the available measures do not go beyond incentives. The development of more sophisticated measures that incorporate the impact of incentives on behavioral actions as well as the impact of behavioral actions on welfare is an important step to better predict the potential effects of alternative risk equalization/sharing designs.

To underline the relevance of our work, we reviewed the official ex-ante evaluation framework used in a well-developed policy-context: the regulated health insurance for curative care in the Netherlands. We find that the Dutch framework used from 2017 to 2022 does not consider all potential effects. None of the 14 potential positive effects of risk equalization and risk sharing are explicitly considered in the framework. Moreover, the framework includes a series of inappropriate measures that poorly link to the potential effects, such as the R-squared and mean prediction errors for insurer-portfolios three years ago. We think this is problematic since an incomplete and/or inappropriate set of evaluation measures can lead to biased conclusions about the performance of risk equalization and risk sharing, resulting in suboptimal policy making. For example, by ignoring the 14 potential positive effects of risk equalization and risk sharing, too much weight will be given to the negative effects of risk sharing or endogenous risk adjusters on efficiency. More specifically, such a one-sided view can lead to the conclusion that risk sharing or endogenous risk adjusters reduce efficiency, while the overall effect on efficiency might be positive.

Our observations lead us to two key recommendations, one for researchers and one for policymakers. Our recommendation for researchers is to develop additional meaningful measures for quantifying the expected effects of risk equalization and risk sharing. This requires going beyond under/overcompensation by developing economic models of how incentives feed into behavior and effects, which might strongly depend on the institutional context and thus vary across systems. Existing models (e.g., [45, 52]) provide a strong basis for this line of research. The challenge will be to customize such models to the setting of interest and to build such models for all potential effects of risk equalization and risk sharing. We realize that it might be too ambitious to develop a complete model for each potential effect in each context. In many settings and for many effects, empirical estimations on how insurers and consumers respond to incentives might not be available. In these cases, a first important step is to construct a conceptual ‘blueprint’ of how incentives might feed into behavior and effects.

Our recommendation for policymakers is to critically review and revise their ex-ante evaluation framework in the light of the 22 potential effects identified in this paper. Although our conclusion that the Dutch Evaluation Framework used from 2017 to 2022 is incomplete only holds for the Netherlands (for the indicated period of time), we expect that ex-ante evaluation frameworks used in other countries are incomplete too.^7^ As a first step, it is crucial for policymakers to carefully consider which of the 22 effects are relevant for their insurance system. This depends on system features such as the presence of an insurance mandate, the set of choice options for consumers, and the flexibility for insurers regarding the design and pricing of insurance products. As a second step, it is important to accurately describe the relevant potential effects in the ex-ante evaluation framework. As a third step, it is crucial to select meaningful quantitative measures for indicating the relevant effects. Although the academic literature provides guidance on the choice of quantitative measures, we realize that the choice of measures might be subject to data restrictions. In case there is no data (or no meaningful measure) available to quantify a potential effect, we recommend evaluating that potential effect qualitatively, e.g., via discussions with experts and stakeholders. By taking into account the *full* spectrum of potential effects, policymakers and regulators will be able to make better-informed choices regarding the design of risk equalization and risk sharing schemes.

## Data Availability

No datasets were generated or analysed during the current study.

## Footnotes

1 In this paper we restrict our consideration of the potential effects of risk selection to effects that directly relate to the basic health insurance.

2 In the presence of moral hazard, sorting of consumers into plans with less comprehensive coverage can lead to a reduction of total healthcare spending. In that case, the *net* welfare effect of the distortion of consumer sorting due to selection-driven premium variation is not clear. On the one hand, there might be a forgone welfare gain since some consumers do not sort into the plan that provides them the highest benefits over costs. On the other hand, if this leads to a reduction in moral hazard, there might also be a welfare gain.

3 In a voluntary insurance market, the possibility for insurers to design products for low-risk consumers might help encourage enrollment. This might be particularly beneficial when affordability is an issue, and healthcare service utilization (regardless of risk status) is primarily concentrated among wealthier consumers. In such a scenario, permitting insurers to introduce a “catastrophic risk” plan targeted at low-income consumers might enhance access and fairness of the healthcare system. As we will explain in Sect. 2.2, risk equalization and risk sharing affect premiums of these products and might influence enrollment, either positively or negatively.

4 Van Kleef et al. [5] find that the prospective risk equalization model used in the Netherlands in 2016 has an R-squared and CPM of 0.3 and reduces predictable profits/losses for subgroups of interest by > 80%. Although the authors do not simulate the effects of a payment system that solely consists of 50% proportional risk sharing, it can be analytically argued that such a system comes with an R-squared of 0.75, a CPM of 0.50 and a reduction of predictable profits/losses for subgroups of interest by 50% (assuming that premiums are community-rated).

5 As discussed at the end of Sect. 2, *estimation* of risk equalization models is typically based on observed differences in healthcare expenditures among risk types, assuming that these differences are genuinely attributable to “true health risk”. The same is true for the *evaluation* of risk equalization models. There are various scenarios in which spending variation is also attributable to differences in “efficiency” across insurers and providers and “under/overutilization” by consumers. Although we do not elaborate on this methodological issue here, we think this issue should be carefully considered when estimating and evaluating risk equalization models.

6 In the winter of 2022/2023, the Evaluation Framework was revised, partly in response to the analysis in this paper.

7 Although the revised version of the Dutch Evaluation Framework includes a broader set of meaningful measures than the previous version, most of the shortcomings identified in Sect. 4 of this paper still hold.
